# Anti-Freezing Eutectogel-Based TENG for Ocean Wave Sensing at Low Temperature

**DOI:** 10.3390/mi17070873

**Published:** 2026-07-22

**Authors:** Siyao Luan, Guoqing Ren, Jinghao Liu, Jiru Xian, Xin Ma, Xiaoyi Li

**Affiliations:** 1 College of Materials Science and Engineering, Ocean University of China, Qingdao 266100, China; 23170001063@stu.ouc.edu.cn (S.L.); 21251713061@stu.ouc.edu.cn (J.L.); 2 Key Laboratory of Physical Oceanography, MOE. China, Ocean University of China, Qingdao 266100, China; renguoqing@ouc.edu.cn (G.R.); maxin@ouc.edu.cn (X.M.)

**Keywords:** eutectogel, self-powered sensing, triboelectric nanogenerator, wave sensing, low temperature

## Abstract

Accurate ocean wave sensing in polar and other low-temperature marine environments is of great significance for marine environmental observation, climate research, and navigation safety. However, conventional wave sensors rely on external power supplies and suffer from poor stability under low-temperature and high-salinity conditions, making long-term self-powered waves sensing a significant challenge. Herein, a highly stable composite eutectogel electrode is developed by integrating sodium lignosulfonate, Fe^3+^ crosslinking, Zn^2+^-carboxylate coordination interactions, and a choline chloride/urea deep eutectic solvent (DES). The DES effectively suppresses solvent crystallization and endows the gel with excellent low-temperature tolerance, while the synergistic effect of metal coordination and multiple non-covalent interactions constructs a robust ion-conducting network with enhanced structural stability. Furthermore, eutectogel-based composite electrode architecture is designed to improve electrical conductivity and charge collection efficiency, thereby enabling stable electrical output under harsh marine conditions. Based on the as-prepared eutectogel electrode, a self-powered solid–liquid triboelectric nanogenerator is fabricated for ocean wave-motion sensing. The device can detect the wave amplitude, with an accuracy of 0.2 cm, and sense the frequency of waves ranging from 0.2 Hz to 1.6 Hz. More importantly, the SL-TENG exhibits excellent environmental adaptability, operating reliably in 3.5 wt% simulated seawater and at 0 °C. The current retention ratio reaches approximately 91% at 0 °C, which is significantly higher than that of the hydrogel-based device (≈6%). The remarkably low-temperature and salt-tolerant performance originates from the stable ion-transport network and anti-freezing characteristics of the eutectogel electrode. This work provides an effective strategy for constructing environmentally resilient eutectogel-based triboelectric devices and offers a promising route toward self-powered wave sensing systems for long-term deployment in harsh marine environments.

## 1. Introduction

Accurate wave sensing is of fundamental importance for marine environmental observation, ocean engineering, navigation safety, and polar scientific exploration. Wave parameters not only reflect ocean dynamic processes but also serve as key indicators for ocean-atmosphere interactions, marine environmental assessment, and navigation safety evaluation [1,2,3]. Particularly in polar and high-latitude regions, where environmental conditions are harsh and replacing the sensor battery is difficult, the development of reliable wave-sensing systems capable of long-term stable operation is highly desirable. Conventional wave-sensing systems rely on batteries or external power supplies, resulting in challenges such as limited energy replenishment, high maintenance costs, and restricted operational lifetimes in remote marine environments. Furthermore, prolonged exposure to low-temperature conditions can deteriorate the electrical and mechanical properties of sensing components, leading to reduced signal stability and measurement accuracy. These challenges are particularly severe in polar marine environments, where low temperatures and high salinity coexist. Therefore, the development of self-powered wave sensors with excellent environmental adaptability and long-term operational stability has attracted increasing research interest [4,5,6].

Triboelectric nanogenerators (TENGs) can directly convert mechanical stimuli into electrical signals through the coupling effects of contact electrification and electrostatic induction, attracting significant attention as self-powered sensing platforms [3,4,5]. Owing to their simple device architecture, low cost, broad material selection and excellent responsiveness to low-frequency mechanical motions, TENGs have been widely investigated in environmental sensing, motion sensing, and marine information detection [7,8,9,10]. Since ocean waves inherently exhibit low-frequency oscillatory characteristics, self-powered wave sensors utilize the corresponding electrical response of TENGs as sensing signals, enabling direct sensing of wave characteristics without additional power consumption [2,11]. Nevertheless, one of the key factors limiting their practical deployment in harsh marine environments is the environmental adaptability of conductive electrode materials [12,13]. Under low-temperature conditions, flexible conductive materials are prone to embrittlement, restricted ion transport, and conductivity deterioration, while high-salinity seawater environments may further affect the stability of interfacial charge transfer, ultimately resulting in degraded sensing performance [14]. Conductive hydrogels have been extensively employed as flexible electrodes in TENGs owing to their excellent flexibility, stretchability, and ionic conductivity [12,15]. Among them, poly (acrylic acid)-based hydrogels have attracted considerable attention because of their facile fabrication, abundant functional groups, and tunable network structures [16]. However, conventional hydrogel systems contain a large amount of free water, which readily freezes under low-temperature conditions, significantly hindering ion transport and disrupting the polymer network structure [14]. In addition, prolonged exposure to air can induce dehydration and shrinkage caused by water evaporation, leading to deterioration of both electrical and mechanical properties. These shortcomings severely restrict their applicability in marine wave sensors intended for long-term operation in low-temperature environments.

To overcome these limitations, eutectogels based on deep eutectic solvents (DESs) have attracted considerable attention owing to their low freezing points, low volatility, and excellent ionic conductivity [7,17,18]. The abundant hydrogen-bonding interactions within DESs can effectively suppress solvent crystallization and evaporation, thereby endowing eutectogels with outstanding anti-freezing capability and environmental stability. For example, Yang et al. developed a zwitterionic eutectogel with enhanced ionic conductivity and excellent self-healing capability, maintaining stable electrical output performance over a wide temperature range [19]. Li et al. reported a biomass-derived conductive eutectogel featuring high stretchability, self-healing capability, and wide-temperature adaptability through the incorporation of multiple dynamic hydrogen-bonding interactions [7]. Furthermore, Sun et al. proposed an internal-external dual-reinforcement strategy that significantly improved the mechanical robustness and environmental tolerance of eutectogels [18]. These works demonstrate the great potential of eutectogels for flexible electronics and self-powered sensing under extreme environmental conditions [7,19].

However, most reported eutectogel electrodes have primarily focused on improving anti-freezing performance, mechanical robustness, or self-healing capability [7,18]. Studies targeting long-term wave-sensing applications in complex marine environments remain relatively underexplored, especially under the combined effects of low temperature and high salinity. Herein, we report a self-powered SL-TENG based on a dual-ion-coordination eutectogel designed for reliable wave sensing in low-temperature marine environments. Unlike previous eutectogel systems that primarily rely on hydrogen-bond regulation or single dynamic interactions, the proposed eutectogel integrates Fe^3+^/Zn^2+^ dual-ion coordination with a choline chloride/urea deep eutectic solvent to simultaneously reinforce structural stability and maintain efficient ion-transport pathways. Specifically, Fe^3+^ participates in coordination interactions with sodium lignosulfonate and polymer chains, while reversible Zn^2+^-carboxylate coordination provides dynamic physical crosslinking, enabling synergistic enhancement of structural robustness and ionic transport. This dual-ion strategy, together with the DES-regulated ion-conducting matrix, allows the device to operate stably under the coupled challenges of low temperature and high salinity. As a result, the SL-TENG achieves quantitative self-powered wave sensing with a wave-amplitude resolution of 0.2 cm and stable frequency detection over 0.2–1.6 Hz, even in 3.5 wt% simulated seawater at 0 °C. This work provides an effective strategy for developing environmentally resilient eutectogel-based self-powered sensors for long-term wave sensing in harsh marine environments.

## 2. Materials and Methods

### 2.1. Materials and Chemicals

Sodium lignosulfonate was purchased from Shanghai Bide Pharmatech Co., Ltd., Shanghai, China. Acrylic acid (AA), urea, choline chloride (ChCl), ferric chloride hexahydrate (FeCl_3_·6H_2_O), and ammonium persulfate (APS) were obtained from Shanghai Macklin Biochemical Co., Ltd., Shanghai, China. Anhydrous zinc chloride (ZnCl_2_) was purchased from Shanghai Aladdin Biochemical Technology Co., Ltd., Shanghai, China. All chemicals were used as received without further purification. Deionized water (DW) was obtained from an ultrapure water purification system and used in all experiments.

### 2.2. Preparation of Deep Eutectic Solvent

The deep eutectic solvent (DES) was prepared by a heating-stirring method. Choline chloride (ChCl) and urea were mixed at a molar ratio of 1:2 and heated at 80 °C under magnetic stirring (300 rpm) for 2 h until a transparent and homogeneous liquid was obtained. The resulting DES was naturally cooled to room temperature (25 °C) and stored in a sealed container for subsequent use.

### 2.3. Preparation of Hydrogels and Eutectogels

Solution A was prepared by dissolving 0.012 g of sodium lignosulfonate, 0.32 g of ferric chloride hexahydrate (FeCl_3_·6H_2_O), and 0.40 g of anhydrous zinc chloride (ZnCl_2_) in 16 mL of deionized water (DW) under magnetic stirring until a clear and homogeneous precursor solution was obtained.

Solution B was prepared by adding 10.8 mL of acrylic acid (AA) into 16 mL of deionized water (DW), followed by the addition of 0.10 g of ammonium persulfate (APS) as the initiator. The mixture was magnetically stirred until a homogeneous solution was formed.

Subsequently, Solutions A and B were thoroughly mixed and poured into a rectangular mold. The resulting precursor solution was allowed to undergo free-radical polymerization at room temperature to form a hydrogel.

**Figure 1:**
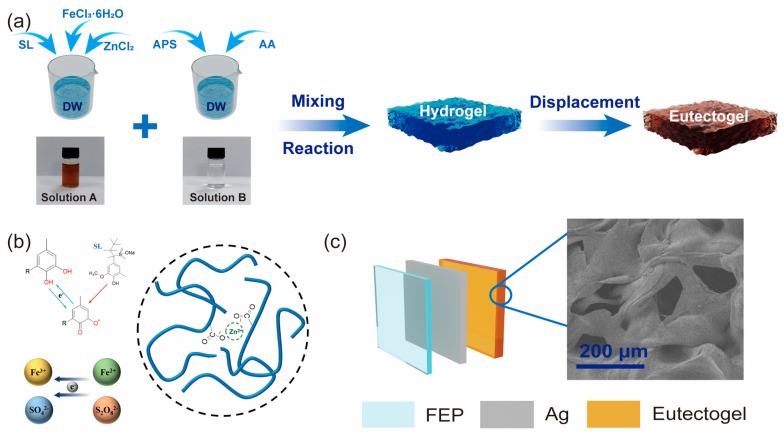
Preparation, structural design, and interaction mechanism of the eutectogel-based SL-TENG. (**a**) Schematic illustration of the two-step preparation process of the eutectogel. (**b**) Schematic representation of the multiple interaction networks within the eutectogel. (**c**) Schematic configuration of the SL-TENG device based on the eutectogel electrode and SEM image of the eutectogel morphology.

The eutectogel was fabricated through a solvent replacement process, as illustrated in Figure 1a. After being removed from the mold, the obtained hydrogel was immersed in the pre-prepared deep eutectic solvent (DES) for 6 h. During this process, the free water within the hydrogel network was gradually replaced by the DES, leading to the formation of a stable DES-rich network. The sample was then removed from the DES, and excess solvent on the surface was carefully wiped off to obtain the Fe^3+^/Zn^2+^ dual-ion crosslinked eutectogel, denoted as eutectogel.

### 2.4. Fabrication of the TENG

The fabricated eutectogel was first mounted onto a glass substrate. A conductive silver layer was then uniformly deposited on the eutectogel surface to serve as the electronic conductive component, while an aluminum wire was embedded at the interface between the silver layer and the eutectogel as the external electrode. After the silver layer was completely cured, a fluorinated ethylene propylene (FEP) film was attached to its surface and employed as the triboelectric layer. Subsequently, the entire device was encapsulated by sealing its edges, as shown in Figure S10, to improve environmental resistance and long-term operational stability. The resulting device, consisting of an Ag/eutectogel composite electrode with coupled electronic and ionic conduction pathways, was used as a fully encapsulated triboelectric nanogenerator (TENG).

### 2.5. Characterization and Measurements

The microstructure and pore morphology of the freeze-dried hydrogel were examined using scanning electron microscopy (SEM, ZEISS GeminiSEM 300, Carl Zeiss Microscopy GmbH, Jena, Germany). The chemical structures and functional group variations were analyzed by Fourier transform infrared (FTIR) spectroscopy (Nicolet iS20, Thermo Scientific, Madison, WI, USA). Raman spectroscopy was employed to investigate molecular interactions and hydrogen-bonding variations (LabRAM HR Evolution, HORIBA, Kyoto, Japan), enabling the evaluation of changes in the internal chemical environment of the eutectogel after DES incorporation. Tensile tests and sticking tests were conducted to evaluate the mechanical robustness and interfacial adhesion capability of the eutectogel. The solvent retention capability was evaluated by monitoring the weight changes in samples before and after environmental exposure. The mass changes in the samples were periodically monitored at room temperature. The solvent retention capability was further assessed by comparing the sample weights before and after freeze-drying. Differential scanning calorimetry (DSC) was conducted to analyze the thermal response behavior of the eutectogel system, thereby evaluating its thermal stability.

The electrical performance of the triboelectric nanogenerator (TENG) was characterized by periodically immersing and withdrawing the device in water using a motor-driven setup to simulate wave-induced mechanical excitation. The device was mounted on a motor-driven linear stage and operated at controlled frequencies to achieve repetitive solid–liquid contact and separation. The open-circuit voltage (Voc), short-circuit current (Isc), and transferred charge (Qsc) were measured using an electrometer and recorded accordingly.

The sensing performance of the SL-TENG was systematically evaluated under different operating conditions, including varying excitation frequencies and vibration amplitudes. Particular attention was paid to its response under low-frequency and small-amplitude excitations to assess its capability for detecting weak wave motions. In addition, the sensing behavior of the device was investigated in 3.5 wt% simulated seawater and at 0 °C to evaluate its environmental adaptability under marine-like conditions. A comparative study between the composite eutectogel-based device and a conventional hydrogel-based counterpart was further conducted to reveal the influence of electrode materials on sensing stability and environmental tolerance under temperature variation.

## 3. Results and Discussion

### 3.1. Formation Mechanism of the Hydrogel and Eutectogel

As shown in Figure 1a, the eutectogel was prepared via a two-step strategy. In the first step, solution A containing sodium lignosulfonate, FeCl_3_·6H_2_O, and ZnCl_2_ was mixed with solution B composed of acrylic acid (AA) and ammonium persulfate (APS), followed by in situ free-radical polymerization at room temperature to form a hydrogel network. In the second step, the hydrogel was subjected to solvent exchange, during which the internal water was progressively replaced by a deep eutectic solvent (DES), yielding the final eutectogel [20,21].

As illustrated in Figure 1b, during the polymerization process, the phenolic hydroxyl groups in sodium lignosulfonate can undergo a redox reaction with Fe^3+^, leading to the partial reduction of Fe^3+^ to Fe^2+^ [8,22]. The interaction between sodium lignosulfonate and Fe^3+^ may facilitate the redox-mediated polymerization process, contributing to the formation of the PAA network [22]. As polymerization proceeds, abundant carboxyl groups in the polymer chains are partially deprotonated to form carboxylate groups (-COO^−^), which provide coordination sites for subsequent metal-ion coordination interactions. Meanwhile, Zn^2+^ can coordinate with the oxygen atoms of carboxylate groups in the PAA chains, forming dynamic metal-ligand coordination interactions that serve as reversible physical crosslinking points within the polymer network [23,24]. Such reversible coordination interactions improve network stability and provide structural adaptability under external stimuli.

After polymerization, the water within the hydrogel was replaced by a deep eutectic solvent (DES) through a solvent exchange process [7,17]. Owing to the abundant hydrogen-bond donors and acceptors in the DES, multiple intermolecular hydrogen-bonding interactions can be formed with the carboxyl, hydroxyl, and other polar functional groups along the polymer chains [25]. As a result, the original water-mediated hydrogen-bonding network within the hydrogel is reconstructed. The formation of multiple hydrogen-bonding interactions between the DES and polymer chains further strengthens the interchain interactions, leading to a more stable three-dimensional network structure with enhanced structural integrity [26].

Accordingly, the resulting eutectogel consists of a covalent poly(acrylic acid) (PAA) network, dynamic Zn^2+^-carboxylate coordination interactions, Fe^3+^-mediated coordination interactions with sodium lignosulfonate (SL), and a DES-induced hydrogen-bonding network, as illustrated in Figure 1b [11]. The synergistic interactions among these components construct a robust three-dimensional network, providing the structural foundation for the enhanced mechanical stability and antifreezing performance of the eutectogel in flexible electronic applications. To further reveal the interconnected network morphology, scanning electron microscopy (SEM) characterization was performed. As shown in Figure 1c, the eutectogel exhibits a homogeneous and continuous three-dimensional porous structure, confirming the formation of an interconnected gel framework. This well-developed network facilitates efficient ion migration while maintaining structural integrity under external environmental variations.

The mechanical flexibility and adhesion capability of the eutectogel were further evaluated. As shown in Figure S7a–c, the eutectogel exhibits excellent stretchability and recovery ability while maintaining structural integrity after deformation. These properties can be attributed to the dynamic and reversible crosslinking interactions within the network, which enable effective stress dissipation and mechanical recovery. Moreover, the eutectogel exhibits strong adhesion to various substrates, including metal, plastic, and glass (Figure S8a–c). The abundant polar groups (e.g., carboxyl and hydroxyl groups) provide multiple interfacial interactions, such as hydrogen bonding, coordination, and electrostatic interactions, enabling stable adhesion. The combination of flexibility and adhesion performance improves the structural stability and integration capability of the eutectogel electrode in flexible electronic devices.

### 3.2. Raman and FTIR Analysis

To further investigate the evolution of intermolecular interactions after DES incorporation, Raman spectroscopy and Fourier transform infrared (FTIR) analysis were conducted on both the hydrogel and eutectogel.

**Figure 2:**
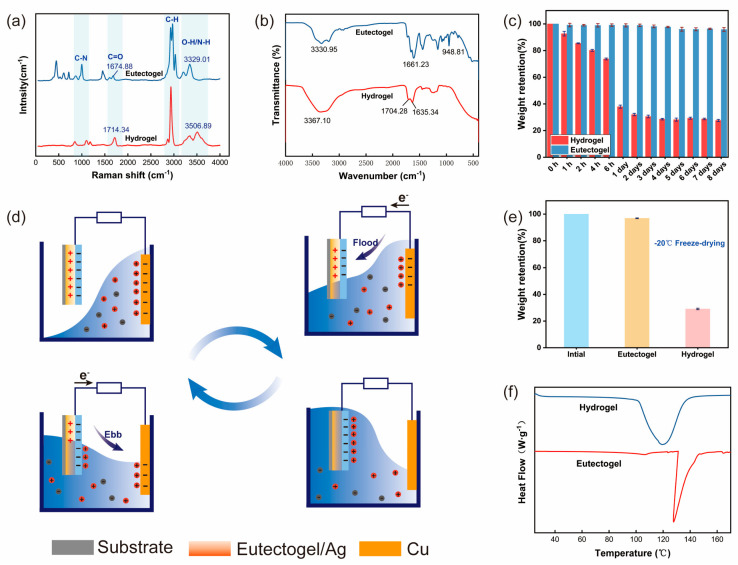
Structural characterization, environmental stability, and working mechanism of the eutectogel-based SL-TENG. (**a**) Raman spectra of the hydrogel and eutectogel. (**b**) FTIR spectra of the hydrogel and eutectogel. (**c**) Weight retention comparison of the hydrogel and eutectogel at room temperature. (**d**) Schematic illustration of the working mechanism of the SL-TENG based on solid–liquid contact electrification and electrostatic induction. (**e**) Weight retention of the hydrogel and eutectogel after freeze-drying treatment at −20 °C. (**f**) DSC curves of the hydrogel and eutectogel.

As shown in Figure 2a, the hydrogel exhibits a characteristic peak at 1714.34 cm^−1^, corresponding to the C=O stretching vibration of carboxyl groups [27]. In contrast, this peak shifts to 1674.88 cm^−1^ in the eutectogel. Such a red shift in the carbonyl stretching band suggests a reduced electron density around the C=O bond, which typically arises from its involvement in strong intermolecular interactions, particularly hydrogen bonding with DES components (e.g., urea and choline chloride) [28,29]. This interaction weakens the C=O bond strength, resulting in a lower vibrational frequency [30]. In addition, enhanced absorption features in the C-N vibration region are observed in the eutectogel, which can be attributed to the incorporation of urea and choline chloride, indicating their effective participation in the polymer-associated interaction network. Meanwhile, the broad O-H/N-H stretching band shifts from 3506.89 cm^−1^ in the hydrogel to 3329.01 cm^−1^ in the eutectogel, accompanied by significant peak broadening [8,22]. This red shift and broadening are characteristic signatures of strengthened and more diverse hydrogen-bonding interactions, reflecting the formation of a more complex hydrogen-bonded supramolecular network involving DES species, polymer chains, and ionic groups [17,31].

Collectively, these spectral changes confirm that DES molecules are not merely physically dispersed within the gel matrix but actively participate in the construction of an extended hydrogen-bonding interaction network with the polymer backbone, thereby reconstructing the internal intermolecular architecture of the gel [32].

Further FTIR analysis was conducted to investigate variations in the functional group environments within the gel networks. As shown in Figure 2b, the hydrogel exhibits a broad absorption band at 3367.10 cm^−1^, which is assigned to the stretching vibrations of O-H and N-H groups arising from water molecules and polymer chains [8]. The characteristic peaks at 1704.28 cm^−1^ and 1635.34 cm^−1^ are attributed to carbonyl-related vibrations of carboxyl groups within the polymer backbone. In contrast, for the eutectogel, the O-H/N-H stretching band shifts to 3330.95 cm^−1^, accompanied by a noticeable broadening, while the carbonyl-related bands shift to 1661.23 cm^−1^ [22]. Such red shifts in both O-H/N-H and C=O vibrations indicate a decrease in bond vibrational energy, which typically arises from the involvement of these functional groups in stronger and more diverse hydrogen-bonding interactions. Specifically, the C=O groups of the polymer chains and the O-H/N-H species derived from the choline chloride/urea DES act as hydrogen-bond acceptors and donors, respectively, forming an extended intermolecular hydrogen-bonding network [12,13].

Similar spectral shifts have been widely reported in choline chloride/urea-based deep eutectic solvent systems and are generally associated with the reconstruction of hydrogen-bonding interaction networks [13,31]. Therefore, these results suggest that the DES is not merely physically dispersed within the polymer matrix, but actively participates in the formation of a dynamic supramolecular hydrogen-bonded network, thereby significantly altering the intermolecular interaction environment of the gel.

The Raman and FTIR results complement each other, confirming that the DES has been successfully incorporated into the polymer network and forms stable intermolecular interactions with polymer chains through hydrogen bonding. Meanwhile, the dynamic coordination between Zn^2+^ and carboxyl groups remains preserved, and together with the DES-induced hydrogen-bonding network, constructs a multiscale noncovalent interaction system within the eutectogel [11,22]. Such a synergistic network structure effectively enhances the structural stability of the gel and endows the material with excellent environmental stability and low-temperature adaptability.

### 3.3. Structural Design and Environmental Stability of the TENG

As shown in Figure 1c, a solid–liquid contact triboelectric nanogenerator (SL-TENG) based on a eutectogel composite electrode was constructed. The device consists of a glass substrate, a eutectogel composite electrode layer, and a fluorinated ethylene propylene (FEP) triboelectric layer [33,34]. The eutectogel composite electrode serves as the functional sensing component, providing efficient ion transport and interfacial charge regulation, while simultaneously ensuring reliable charge collection and signal transmission during device operation [35]. Electrical signals generated by the contact electrification process are collected through an external circuit for subsequent sensing and sensing applications. The eutectogel possesses a homogeneous and continuous three-dimensional crosslinked network structure. This interconnected architecture provides continuous pathways for ion migration and facilitates uniform charge distribution throughout the gel matrix. Such structural characteristics contribute to stable interfacial charge generation and signal transduction, which are essential for maintaining reliable sensing performance under dynamic operating conditions [36]. These features demonstrate the suitability of the eutectogel composite electrode for self-powered ocean-wave sensing applications.

As illustrated in Figure 2d, the SL-TENG operates based on the coupled effects of solid–liquid contact electrification and electrostatic induction, enabling the conversion of wave-induced mechanical stimuli into measurable electrical signals for self-powered sensing [37,38,39,40]. Charge redistribution occurs within the composite electrode due to the difference in interfacial charge transfer behavior between the FEP layer and the eutectogel-based composite electrode. The “+” and “–“ symbols shown in Figure 2d represent the instantaneous polarization states generated during operation rather than permanently charged regions or intrinsic polarity of the electrode materials. Specifically, the “–“ region corresponds to electron accumulation, whereas the “+” region represents an electron-deficient state induced by charge transfer and electrostatic induction. As wave motion causes the seawater level to rise (Flood process), the contact area between seawater and the FEP surface gradually increases, resulting in the redistribution of interfacial charges and a variation in the local electric potential. Driven by this potential difference, electrons flow through the external circuit, generating an electrical response signal. When the seawater level reaches its maximum position and subsequently recedes (Ebb process), the solid–liquid contact area decreases and the induced electric field reverses. Consequently, electrons flow in the opposite direction, producing a reverse electrical signal. During the periodic rise and fall of seawater, electrons continuously shuttle through the external circuit, generating alternating electrical signals that accurately reflect variations in wave motion [39]. The eutectogel composite electrode provides efficient ion transport and stable interfacial charge regulation, facilitating reliable signal generation and transmission. Such characteristics are advantageous for achieving stable and continuous self-powered ocean-wave sensing in complex marine environments.

Beyond the working mechanism, the environmental stability of the electrode material is another crucial factor determining long-term sensing reliability. Therefore, the mass-retention behavior of hydrogel and eutectogel was further investigated. As shown in Figure 2c, the hydrogel and eutectogel exhibit distinctly different mass-retention behaviors under ambient conditions. The hydrogel undergoes a rapid decrease in mass due to continuous water evaporation [31,40]. In contrast, the eutectogel maintains a substantially higher mass-retention rate throughout the testing period, demonstrating excellent solvent-retention capability and structural stability. This behavior can be primarily attributed to the low volatility of the choline chloride/urea deep eutectic solvent (DES) and the dense hydrogen-bonding network formed between the DES components and polymer chains, which effectively suppresses solvent migration and evaporation [21,41]. Previous studies have reported that eutectogels based on DES systems can maintain long-term structural stability and low solvent loss even under harsh environmental conditions, owing to their intrinsically low vapor pressure and strong intermolecular interactions [42]. Furthermore, the abundant dynamic hydrogen-bonding interactions established between the DES and polymer chains help stabilize the ionic transport environment while inhibiting solvent diffusion and network relaxation [7,13]. Consequently, the eutectogel exhibits enhanced environmental stability and structural integrity, which are essential for maintaining reliable electrical output during long-term operation.

The antifreezing capability of gel electrodes is crucial for applications in low-temperature marine environments. As shown in Figure 2e, after exposure to −20 °C, the eutectogel retained a considerably higher mass-retention rate, whereas the hydrogel exhibited obvious mass loss. The distinctly different behaviors indicate that the incorporation of a deep eutectic solvent (DES) significantly enhances the low-temperature stability of the gel system. This improvement is primarily attributed to the unique physicochemical characteristics of the choline chloride/urea DES. The extensive hydrogen-bonding network and strong ionic interactions within the DES effectively disrupt the formation of ordered crystalline domains, thereby suppressing solvent crystallization and mitigating freeze-induced structural damage. As a result, the eutectogel maintains the integrity of its polymer network and preserves continuous ion-transport pathways under low-temperature conditions. Previous studies have shown that DES-based eutectogels exhibit excellent antifreezing properties and can operate over a wide temperature range [19,22]. More importantly, such materials demonstrate the ability to maintain structural integrity and interfacial stability under extreme subzero conditions, highlighting their suitability for use in harsh environments. Therefore, the eutectogel developed in this work serves as a stable electrode platform for self-powered wave sensing in low-temperature marine environments.

To further investigate the regulatory effect of the DES on the gel network structure, differential scanning calorimetry (DSC) measurements were performed on both the hydrogel and eutectogel. As shown in Figure 2f, the hydrogel exhibited a broad endothermic peak within the temperature range of approximately 100–140 °C, whereas the eutectogel displayed a more concentrated and sharper endothermic feature centered around 130 °C. The distinct thermal response behaviors of the two gels indicate that the introduction of the DES altered the intermolecular interaction environment within the system. For the hydrogel, the broadened endothermic peak suggests the coexistence of intermolecular interactions with varying strengths and a relatively complex microenvironment, resulting in a continuous thermal response during heating [22]. In contrast, the endothermic peak of the eutectogel shifted toward a higher temperature region and became more concentrated, indicating the formation of a more stable and homogeneous interaction network.

Combined with the FTIR and Raman results, it can be inferred that, after the incorporation of the ChCl/Urea deep eutectic solvent, the hydroxyl groups of choline cations, the amino groups of urea molecules, and Cl^−^ ions can form extensive hydrogen-bonding interactions with the carboxyl groups on the PAA chains, thereby constructing a stable supramolecular network. Previous studies have demonstrated that the complex hydrogen-bonding network within the ChCl/Urea system is a key contributor to its unique thermal behavior and physicochemical properties [12,13]. Therefore, the shift in the thermal transition peak toward a higher temperature in the eutectogel suggests that a greater energy input is required to disrupt this network, reflecting enhanced intermolecular interactions within the system. Furthermore, the more concentrated thermal transition behavior of the eutectogel indicates that the DES participated in the reconstruction of the polymer network, leading to a more homogeneous microstructure. Previous studies have also reported that DESs can act as functional components in gel systems, forming stable hydrogen-bonding networks with polymer chains and endowing the gels with excellent structural stability and environmental adaptability. These observations are consistent with the enhanced hydrogen-bonding interactions revealed by the FTIR and Raman analyses, further confirming the role of the DES in strengthening the intermolecular interaction network within the eutectogel.

Based on the above experimental results, it can be inferred that the DES not only provides an efficient ionic transport medium but also effectively suppresses network relaxation and component migration through the formation of a stable hydrogen-bonding network. As a result, both the structural stability and electrical stability of the gel are significantly enhanced under complex environmental conditions, providing a solid foundation for the long-term stable operation of the SL-TENG in marine environments.

### 3.4. Electrical Output Performance of the SL-TENG

To evaluate the wave-sensing capability of the fabricated SL-TENG, its electrical responses were systematically investigated under varying excitation frequencies, amplitudes, and complex operating conditions. Before evaluating the sensing performance, the effect of electrode composition was investigated by comparing SL-TENG devices equipped with hydrogel and eutectogel composite electrodes. As shown in Figure S5, the eutectogel-based SL-TENG exhibited more stable and distinguishable electrical signals than the hydrogel-based device. This enhancement is attributed to the stable ion-conducting network and improved interfacial charge regulation capability of the eutectogel electrode, which facilitate charge redistribution during solid–liquid contact electrification and enable reliable signal generation [42,43]. These results confirm that the eutectogel composite electrode is crucial for enhancing the sensing stability and accuracy of the SL-TENG under complex marine conditions. Based on this optimized electrode configuration, the wave-sensing performance of the SL-TENG was further evaluated by correlating its electrical responses with different wave excitation parameters.

**Figure 3:**
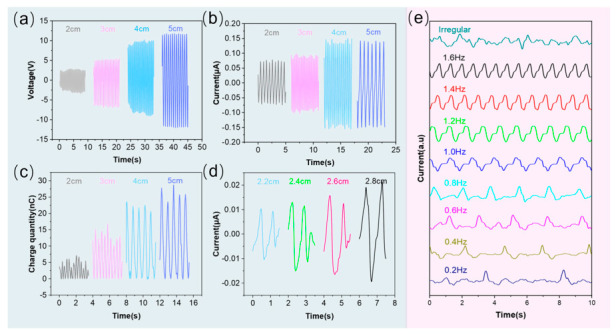
Electrical output performance characterization of the SL-TENG. (**a**–**c**) Amplitude-dependent output signals of the SL-TENG, including open-circuit voltage, short-circuit current, and transferred charge. (**d**) Current response of the SL-TENG to different simulated wave heights. (**e**) Current responses of the SL-TENG under low-frequency wave excitations from 0.2 to 1.6 Hz.

The wave-height sensing capability of the SL-TENG was first evaluated under wave excitations ranging from 2 to 5 cm. As shown in Figure 3a–c, distinct electrical signals were generated under different wave amplitudes, and the corresponding output characteristics varied systematically with wave height. The open-circuit voltage (Voc), short-circuit current (Isc), and transferred charge (Qsc) increased from approximately 3 to 12 V, 0.07 to 0.15 μA, and 6 to 28 nC, respectively, as the wave height increased from 2 to 5 cm. The monotonic variation in these electrical parameters with wave amplitude indicates that the SL-TENG can effectively establish a quantitative correlation between electrical response and wave height. Moreover, stable and repeatable signals were maintained throughout the entire testing range, demonstrating reliable wave-height sensing capability [44,45]. To further assess the resolution of the device toward weak ocean-wave motions, current responses under small wave amplitudes ranging from 2.2 to 2.8 cm were investigated (Figure 3d and Figure S2). Although the difference in wave height was only 0.2 cm between adjacent conditions, distinguishable current signals were still obtained. The signal amplitude exhibited a gradual increase with increasing wave height, indicating that the SL-TENG is capable of detecting subtle wave-height variations and possesses high sensitivity to weak mechanical disturbances. Such characteristics are particularly important for sensing low-energy ocean waves and small-amplitude surface fluctuations.

Furthermore, the frequency-dependent sensing behavior of the SL-TENG was evaluated over a wide frequency range from 0.2 to 1.6 Hz. As shown in Figure 3e and Figure S1, the signal period decreased correspondingly with increasing wave frequency while maintaining good stability and periodicity. Distinguishable current waveforms were obtained throughout the entire frequency range, enabling accurate identification of different wave frequencies. Notably, clear response signals could still be observed at a low frequency of 0.2 Hz, demonstrating excellent low-frequency detection capability.

Overall, the above results demonstrate that the SL-TENG exhibits stable and distinguishable electrical responses under varying excitation frequencies, wave amplitudes, and simulated wave heights. In particular, the device maintains reliable output signals over a broad low-frequency range, enabling effective identification of different wave conditions. These characteristics highlight the excellent wave-sensing capability of the SL-TENG and provide a solid foundation for its application in self-powered ocean-wave sensing and marine environmental sensing.

### 3.5. Polar Marine Environmental Adaptability and Temperature Stability of the SL-TENG

**Figure 4:**
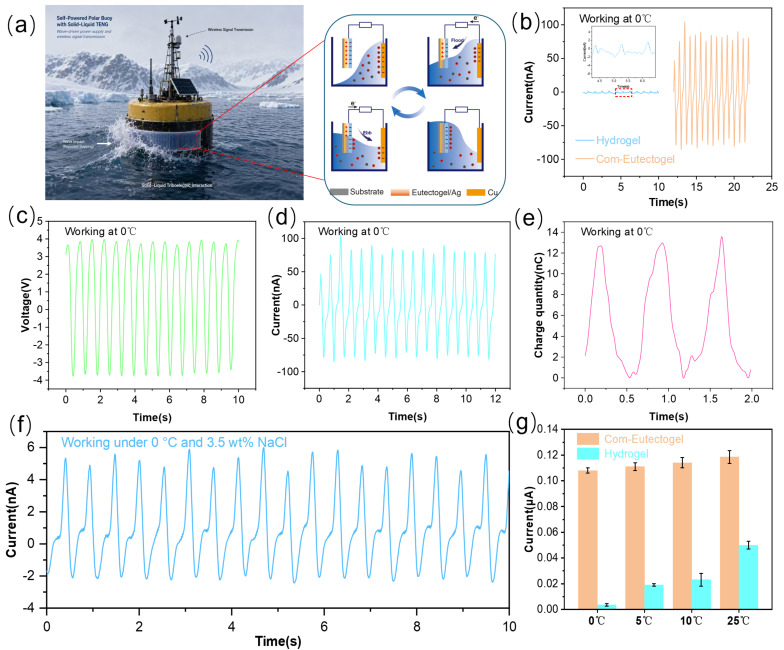
Marine environmental adaptability and temperature stability of the SL-TENG. (**a**) Schematic illustration of a self-powered marine buoy based on the SL-TENG and the corresponding solid–liquid contact electrification mechanism. (**b**) Comparison of the output current of SL-TENGs employing a hydrogel electrode and a composite eutectogel electrode at 0 °C. (**c**–**e**) Open-circuit voltage (Voc), short-circuit current (Isc), and transferred charge (Qsc) of the SL-TENG measured under a 0 °C environment. (**f**) Current signals generated by the SL-TENG operating at 0 °C in 3.5 wt% NaCl solution. (**g**) Comparison of the output current of SL-TENGs based on hydrogel and composite eutectogel electrodes under different temperature conditions.

Polar marine environments are characterized by harsh conditions, including high humidity, high salinity, and low temperatures. Therefore, the development of triboelectric nanogenerators with strong environmental adaptability and stable electrical output is of great importance for reliable ocean sensing in such environments [46]. As illustrated in Figure 4a, the SL-TENG incorporating a composite eutectogel electrode can be integrated into a conceptual self-powered marine buoy system for ocean wave sensing. Under wave excitation, periodic contact and separation between seawater and the triboelectric surface generate corresponding electrical signals. During operation, fluctuations in the seawater level induce the generation and redistribution of interfacial charges at the solid–liquid interface, leading to a potential difference that drives electron flow through the external circuit. The resulting electrical output can effectively reflect variations in wave motion. These characteristics provide a reliable basis for self-powered ocean wave sensing and long-term marine environmental sensing in polar and other low-temperature marine environments [38,41].

To evaluate the low-temperature adaptability of the eutectogel composite electrode, comparative sensing experiments were conducted at 0 °C using SL-TENGs equipped with conventional hydrogel electrodes and eutectogel composite electrodes. As shown in Figure 4b and Figure S4, the hydrogel-based device exhibited significantly reduced and unstable electrical responses under low-temperature conditions, whereas the SL-TENG with the eutectogel composite electrode maintained stable and distinguishable signals. This improved performance is attributed to the introduction of the ChCl/urea deep eutectic solvent (DES), which suppresses solvent crystallization and preserves the flexibility of the gel network under cold conditions.

The low-temperature sensing capability of the SL-TENG was further evaluated at 0 °C. As shown in Figure 4c–e, the device generated stable and repeatable open-circuit voltage (Voc), short-circuit current (Isc), and transferred charge (Qsc) signals, with a voltage response of approximately ±4.0 V. The periodic electrical outputs demonstrate that the solid–liquid triboelectric interface remains effective under near-freezing conditions, confirming the feasibility of the eutectogel-based SL-TENG for self-powered wave sensing in cold marine environments. Furthermore, considering the complex freezing–thawing conditions in marine environments, the stability of the eutectogel electrode was further evaluated through repeated freeze–thaw cycles in 3.5 wt% NaCl solution at 0 °C. As shown in Figure S12, the eutectogel-based SL-TENG maintained a stable electrical response retention after multiple freeze–thaw cycles, demonstrating its excellent anti-freezing capability and environmental adaptability under simulated marine conditions.

The temperature adaptability of the two electrode systems was further compared by analyzing the signal retention under different temperatures (Figure 4g). When the temperature decreased from 25 °C to 0 °C, the eutectogel-based SL-TENG retained approximately 91% ± 0.21% of its initial response, whereas the hydrogel-based device retained only 6% ± 0.16% (n = 5). The results were obtained from five independently fabricated devices, demonstrating the superior low-temperature stability of the eutectogel electrode and its capability to preserve reliable interfacial charge-transfer processes under cold conditions. Additionally, the temperature-dependent resistance and conductivity variations in the hydrogel and eutectogel electrodes were investigated. As shown in Figure S9, the eutectogel electrode exhibited a much smaller resistance increase and higher conductivity retention during cooling from 25 °C to 0 °C compared with the hydrogel electrode. This behavior indicates that the eutectogel effectively maintains ion mobility under low-temperature conditions, benefiting from the DES-regulated solvent environment and the possible contribution of Zn^2+^ coordination interactions to low-temperature ionic conductivity. Similar metal-ion-assisted enhancement of conductive and anti-freezing properties has also been reported in lignin-based hydrogel systems [46]. Furthermore, XRD analysis was conducted to investigate the structural stability of the eutectogel during cooling. As shown in Figure S11, broad diffraction halos were observed at both 25 °C and 0 °C without obvious crystalline peaks, confirming the preservation of an amorphous structure after cooling. The suppressed crystallization behavior provided by the ChCl/urea DES contributes to maintaining the structural stability of the eutectogel under freezing-prone conditions.

Beyond low-temperature tolerance, saline resistance is essential for practical marine sensing applications. Therefore, continuous sensing tests were performed in a simulated seawater environment containing 3.5 wt% NaCl solution at 0 °C. As shown in Figure 4f and Figure S3, the SL-TENG maintained stable and periodic voltage, current, and charge signals without obvious attenuation during saline operation. This result demonstrates that the eutectogel electrode possesses excellent salt tolerance and can sustain reliable sensing performance in high-salinity environments. Furthermore, the long-term operational stability of the SL-TENG was evaluated under combined low-temperature and saline conditions. As shown in Figure S6, the device maintained stable and repeatable sensing signals during continuous operation at 0 °C in 3.5 wt% NaCl solution, with no apparent signal decay or waveform distortion. These results confirm the potential of the SL-TENG for long-term self-powered wave monitoring in harsh marine environments.

Overall, the excellent environmental adaptability of the SL-TENG originates from the stable eutectogel electrode structure enabled by the ChCl/urea DES and dynamic interactions within the polymer network. The DES component suppresses crystallization and improves low-temperature tolerance, while the coordinated network interactions provide mechanical robustness and structural stability. These combined effects enable reliable signal generation and wave sensing under cold and saline marine conditions.

## 4. Conclusions

In this work, a dual-ion-coordinated conductive eutectogel with excellent anti-freezing capability and environmental stability was developed through the synergistic integration of sodium lignosulfonate, Fe^3+^/Zn^2+^ coordination interactions and a choline chloride/urea deep eutectic solvent. The reconstructed dynamic conductive network effectively enhanced ion transport and maintained structural stability in both low-temperature and high-salinity environments. At 0 °C, the eutectogel-based device retained approximately 91% of its room-temperature electrical response, significantly outperforming its hydrogel-based counterpart with only 6%. Benefiting from the excellent environmental adaptability of the eutectogel, the eutectogel-based TENG sensor achieved stable and accurate wave sensing in 3.5 wt% simulated seawater and at low temperatures, with a wave-amplitude detection resolution of 0.2 cm and a frequency sensing range of 0.2–1.6 Hz. Moreover, the device maintained continuous and stable sensing signals under simulated polar seawater conditions, demonstrating its capability for long-term operation in harsh marine environments. These results highlight the effectiveness of eutectogel engineering in overcoming the environmental limitations of conventional hydrogels and establish a practical strategy for realizing accurate self-powered wave sensing in extreme marine environments, which advances ocean-sensing technologies for polar and low-temperature applications.

## Data Availability

Data are available upon request from the authors.

## Supplementary Materials

The following supporting information can be downloaded at https://www.mdpi.com/article/10.3390/mi17070873/s1. Figure S1: Current output signals of the composite eutectogel-based SL-TENG under excitation frequencies: (a) 0.2 Hz. (b) 0.4 Hz. (c) 0.6 Hz. (d) 0.8 Hz; Figure S2: Current output signals of the composite eutectogel-based SL-TENG under different excitation amplitudes corresponding to wave heights: (a) 2.2 cm. (b) 2.4 cm. (c) 2.6 cm. (d) 2.8 cm; Figure S3: Electrical output performance of the composite eutectogel-based SL-TENG in 3.5 wt% NaCl solution (simulated seawater): (a) open-circuit voltage. (b) short-circuit current. (c) transferred charge output; Figure S4: Comparison of the low-temperature electrical output performance of hydrogel- based and composite eutectogel-based SL-TENGs at 0 °C: (a,c,e) voltage, current, and transferred charge outputs of the hydrogel-based device (b,d,f) voltage, current, and transferred charge outputs of the composite eutectogel-based device; Figure S5: Electrical output comparison of the SL-TENG devices based on hydrogel and composite eutectogel electrodes. (a) Open-circuit voltage. (b) Short-circuit current. (c) Transferred charge. Figure S6: Stability of TENG under 0 °C and 3.5 wt% NaCl conditions; Figure S7: Tensile tests of eutectogel. (a–c) The stretching deformation and recovery behavior of the eutectogel; Figure S8: Adhesion tests of eutectogel. (a–c) Adhesion performance of the eutectogel on different substrates; Figure S9: Comparison of the resistance and conductivity of hydrogel and com-eutectogel at 0 °C and 25 °C; Figure S10: The eutectogel-based composite electrode; Figure S11: The eutectogel-based composite electrode. Figure S12. The peak current of com-eutectogel experience repeated freezing–thawing.

## Abbreviations

The following abbreviations are used in this manuscript:

DES	Deep eutectic solvent
SL-TENG	Solid–liquid triboelectric nanogenerator
PAA	Poly (acrylic acid)
ChCl	Choline chloride
APS	Ammonium persulfate
DW	Deionized water
FEP	Fluorinated ethylene propylene
SEM	Scanning electron microscopy
FTIR	Fourier transform infrared
DSC	Differential scanning calorimetry
XRD	X-ray diffraction
