# Supplementary material for: Anti-Freezing Eutectogel-Based TENG for Ocean Wave Sensing at Low Temperature
Source: Micromachines (Basel). 2026 Jul 22;17(7):873. doi: 10.3390/mi17070873 (PMC13413547; doi:10.3390/mi17070873)
Supplement: Supplementary file 1 [file micromachines-17-00873-s001.zip › micromachines-4414098-supplementary.pdf]

Supplementary Materials

Anti-Freezing Eutectogel-Based TENG for Ocean Wave Sensing at Low Temperature

Siyao Luan [1], Guoqing Ren [2], Jinghao Liu [1], Jiru Xian [1,*], Xin Ma [2], Xiaoyi Li [1,*]

1 College of Materials Science and Engineering, Ocean University of China, Qingdao 266100, China; 23170001063@stu.ouc.edu.cn; 21251713061@stu.ouc.edu.cn; 21251713070@stu.ouc.edu.cn; lixiaoyi@ouc.edu.cn

2 Key Laboratory of Physical Oceanography. MOE. China, Ocean University of China, Qingdao 266100, China; renguoqing@ouc.edu.cn; maxin@ouc.edu.cn

* Correspondence:

21251713070@stu.ouc.edu.cn; maxin@ouc.edu.cn; lixiaoyi@ouc.edu.cn

Supplemental Figures

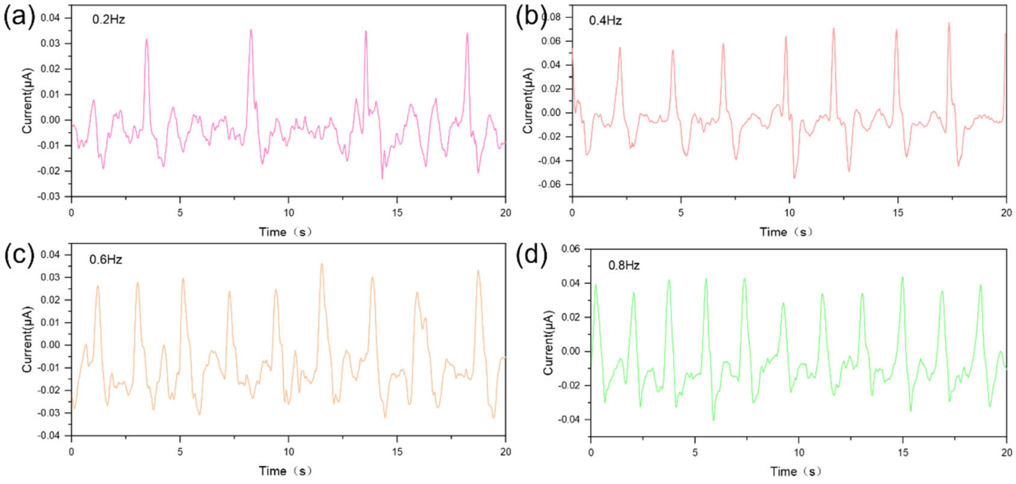

Figure S1. Current output signals of the composite eutectogel-based SL-TENG under excitation frequencies: (a) 0.2 Hz. (b) 0.4 Hz. (c) 0.6 Hz. (d) 0.8 Hz.

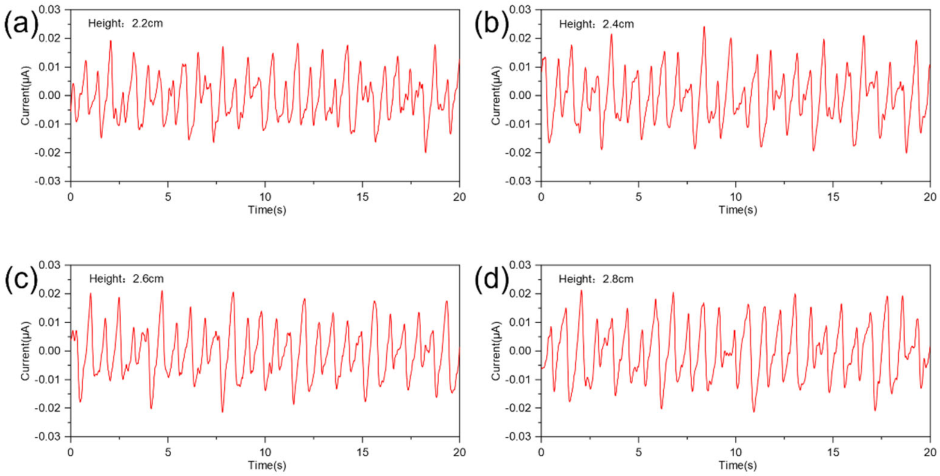

Figure S2. Current output signals of the composite eutectogel-based SL-TENG under different excitation amplitudes corresponding to wave heights: (a) 2.2 cm. (b) 2.4 cm. (c) 2.6 cm. (d) 2.8 cm.

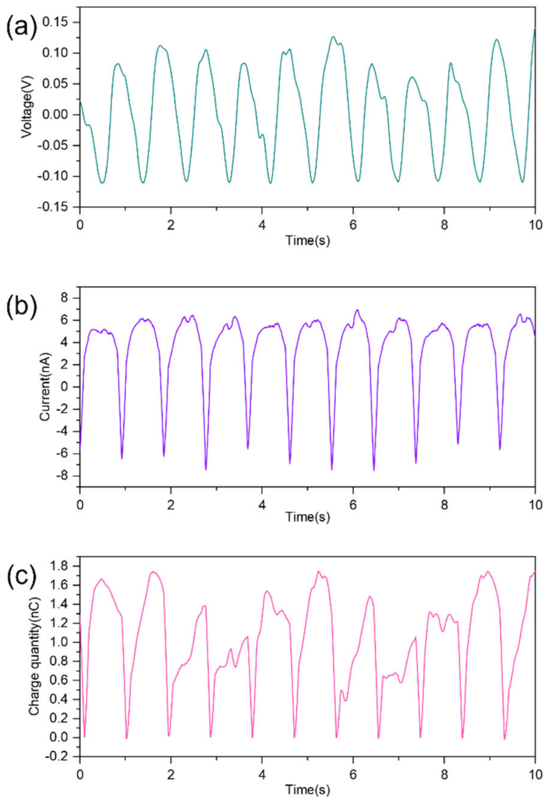

Figure S3. Electrical output performance of the composite eutectogel-based SL-TENG in 3.5 wt% NaCl solution (simulated seawater): (a) open-circuit voltage. (b) short-circuit current. (c) transferred charge output.

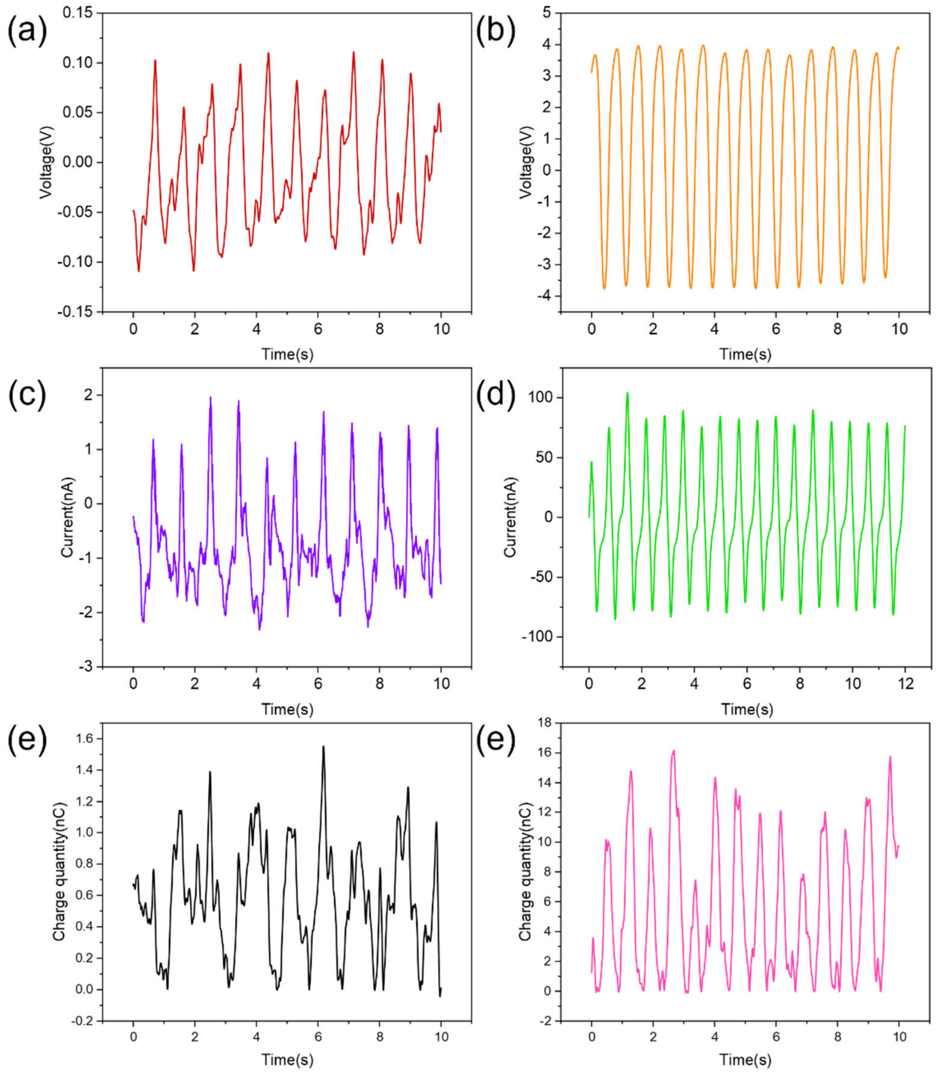

Figure S4. Comparison of the low-temperature electrical output performance of hydrogel-based and composite eutectogel-based SL-TENGs at 0 °C: (a, c, e) voltage, current, and transferred charge outputs of the hydrogel-based device (b, d, f) voltage, current, and transferred charge outputs of the composite eutectogel-based device.

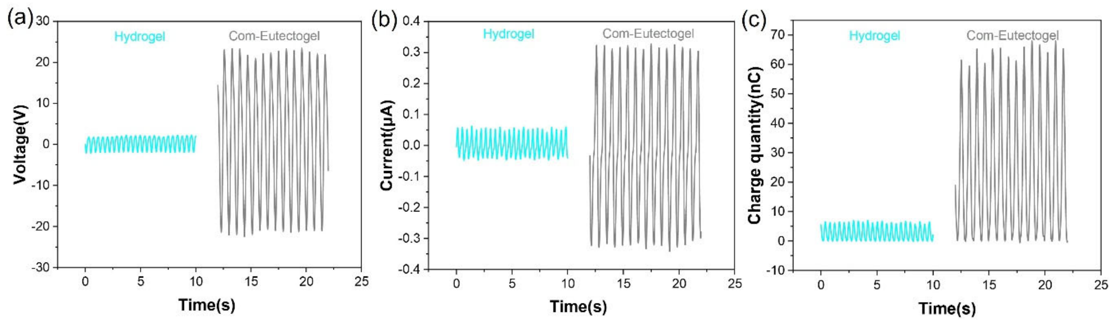

Figure S5. Electrical output comparison of the SL-TENG devices based on hydrogel and composite eutectogel electrodes. (a) Open-circuit voltage. (b) short-circuit current. (c) transferred charge.

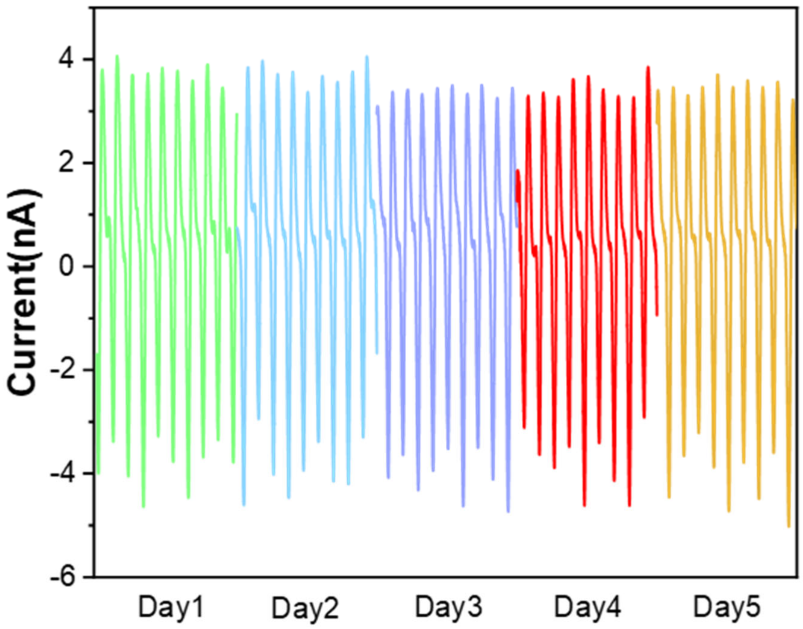

Figure S6. Stability of TENG under 0 °C and 3.5 wt% NaCl conditions.

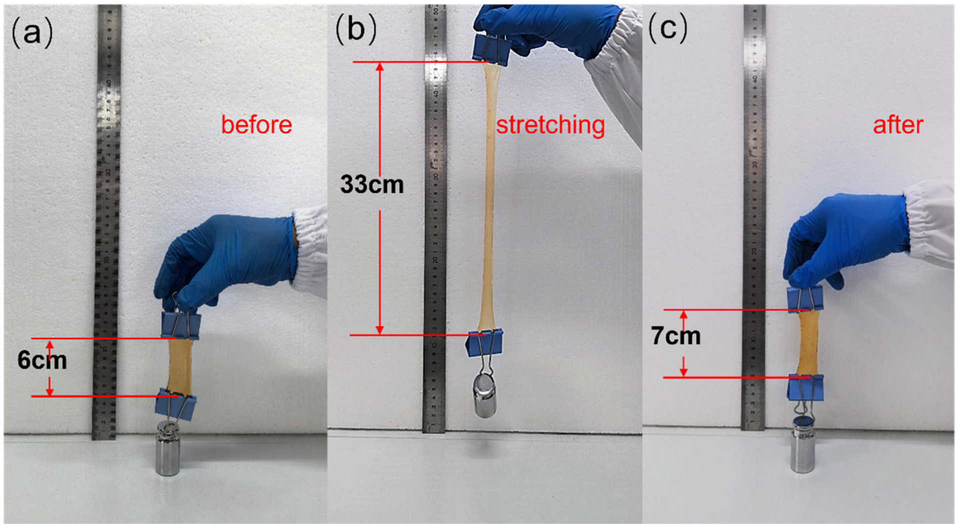

Figure S7. Tensile tests of eutectogel. (**a-c**) The stretching deformation and recovery behavior of the eutectogel.

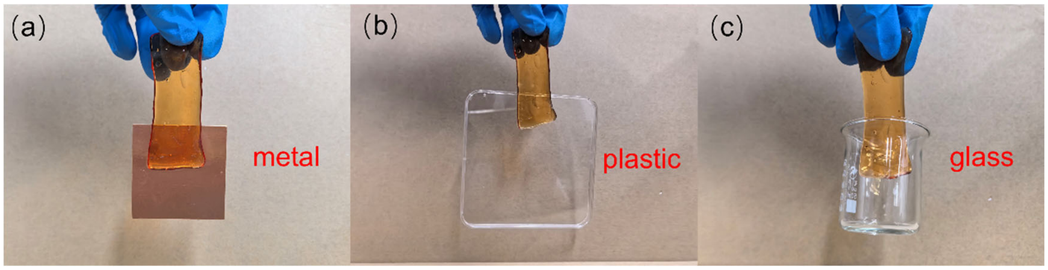

Figure S8. Adhesion tests of eutectogel.(**a-c**) Adhesion performance of the Com-Eutectogel on different substrates.

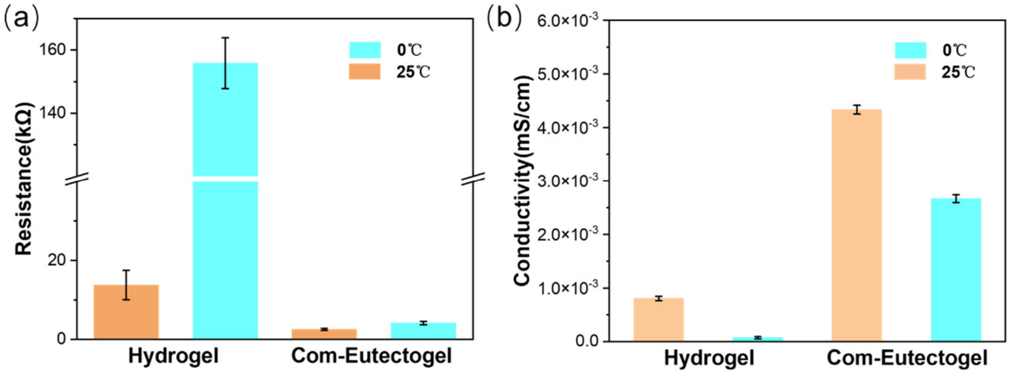

Figure S9. Comparison of the resistance and conductivity of hydrogel and com-eutectogel at 0℃ and 25℃.

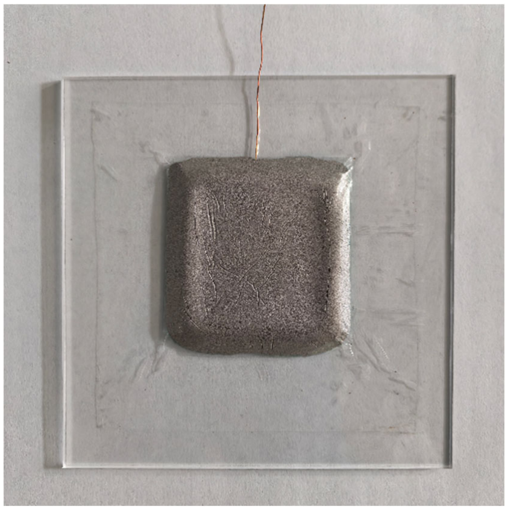

Figure S10. The eutectogel-based composite electrode.

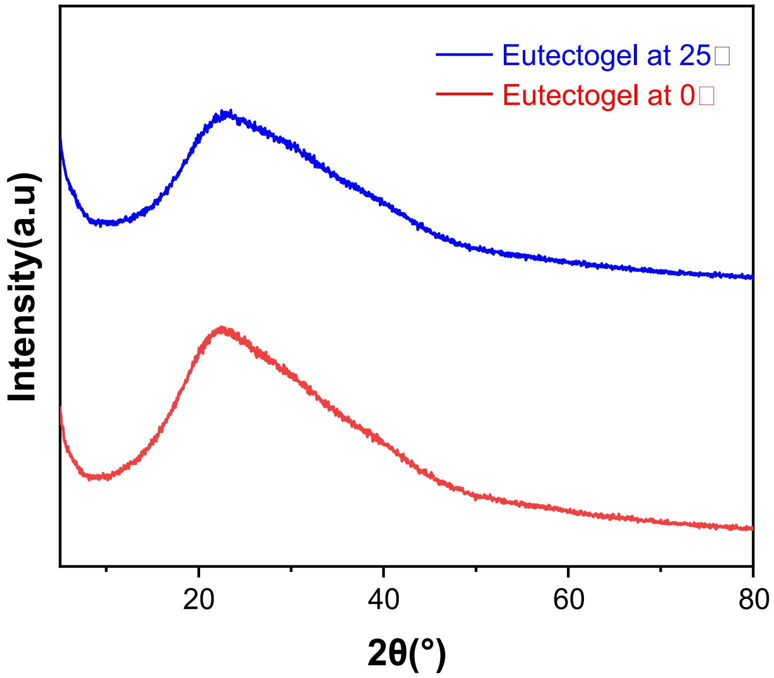

Figure S11. The eutectogel-based composite electrode.

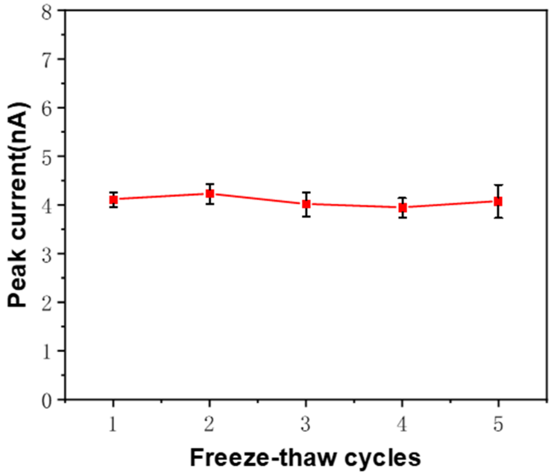

Figure S12. The peak current of com-eutectogel experience repeated freezing–thawing.
